# Beyond Scrubs: Understanding the Root Causes of Violence Against Doctors

**DOI:** 10.7759/cureus.39559

**Published:** 2023-05-27

**Authors:** Priyanshu Jain, Kamaldeep Singh, Shobhit Piplani, Shreya Gulati, Harpreet Kour

**Affiliations:** 1 Medicine, Jawaharlal Nehru Medical College, Belagavi, IND; 2 Cardiology, Government Medical College & Hospital, Chandigarh, IND; 3 Physiology, Jawaharlal Nehru Medical College, Belagavi, IND

**Keywords:** healthcare worker safety, perceived stress among doctors, occupational hazards, patient aggression, workplace violence, workplace safety, workplace violence prevention, violence against doctors

## Abstract

Workplace violence (WPV) against doctors is a growing epidemic in India, with at least two-thirds of doctors facing some form of abuse during their careers. Verbal abuse is common, but doctors are also subjected to brutal attacks that endanger their lives. This review lists abusive incidents reported by the media since 2021. Despite increased respect for healthcare professionals during the COVID-19 pandemic, doctors in India are under significant stress due to inadequate medical infrastructure, mismanagement of young doctors, increasing mistrust between doctors and patients, a shortage of doctors, and overworked healthcare workers, leading to delays in attention and treatment. Additional factors contributing to the situation include the lack of proper insurance coverage, weak primary healthcare with overburdened tertiary care, the lack of an effective grievance redressal system, and the poor state of medical education. To combat this epidemic, collaborative efforts are needed between doctors, hospitals, the government, and society. Improving communication skills and treating patients with empathy are essential for healthcare workers. Meanwhile, hospitals should implement an efficient security system, a transparent billing system, and an active complaint system to prevent incidents. Unbiased reporting and adequate documentation are required to further investigate this occupational health hazard. The government should focus on building better medical facilities and passing a strict law against violence against doctors to ensure the safety of medical professionals. This review presents some solutions, along with the current legal coverage provided to healthcare professionals regarding WPV.

## Introduction and background

In India, the medical community is facing a silent epidemic of violence against doctors. A study by the IMA (Indian Medical Association) discovered that a staggering 82.7% of doctors experience anxiety and stress. Another study published in the Indian Journal of Psychiatry suggests that approximately 75% had encountered some form of violence during their careers [1,2]. Despite the devastating impact of the pandemic on the medical profession, with nearly 2000 doctors lost since 2020, violence against doctors remains a persistent threat [3]. Most of these incidents, ranging from 60% to 70%, take the form of verbal abuse or hostile gestures [4]. Unfortunately, even during the peak of the pandemic, workplace violence (WPV) remained constant, occurring in COVID-designated hospitals, quarantine centers, and even at the residences of medical personnel. However, India's principal public health concern, which has been developing for decades, still has not been addressed. Based on an extensive literature search, this review article examines the violence against doctors in India, the contributing aspects, and possible tactics for avoiding it while demonstrating the gap in the legal measures already available to doctors.

What is workplace violence?

**Figure 1:**
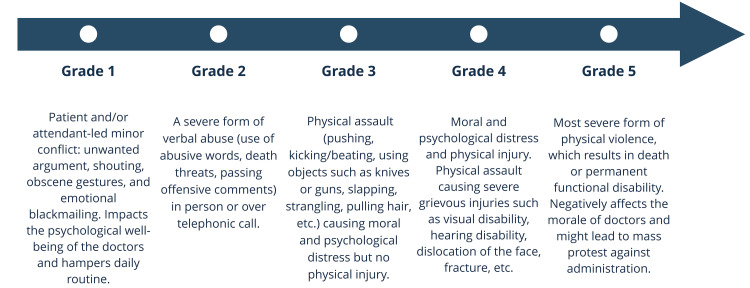
Grades of violence directed at doctors The illustration of the data derived from a study by Kumari et al. [6] has been created by the authors.

According to the World Health Organization (WHO), "incidents where employees are abused, threatened, assaulted, or subjected to offensive behavior in circumstances related to their work, including commuting to and from work, involving an explicit or implicit challenge to their safety, well-being, or health are defined as Workplace Violence (WPV)" [5]. Violence against doctors can be classified into the five grades displayed in Figure 1 [6].

A study published in 2020 on the pattern of WPV against doctors suggests that physicians are often verbally abused (91.2%) and verbally threatened (60.8%) [4]. Non-physical modes of violence are also the most common sort of violence doctors confront globally. Several incidents of similar violence have been documented throughout this pandemic, pointing toward the country's poor public health system (see Appendices for details on major incidents of WPV targeted at doctors from 2021 to 2023, state-wise distribution of WPV against medical personnel, and links to reported and publicized incidents of WPV against doctors).

## Review

Factors contributing to the epidemic of violence against doctors

**Figure 2:**
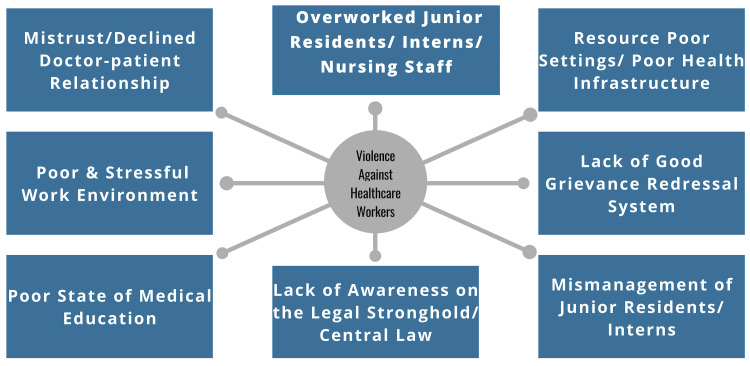
A chart depicting the factors contributing to the WPV epidemic The illustration of this input has been created by the authors.

Violence against doctors is a multifactorial public hazard. In this review, we examined several factors directly or indirectly associated with this epidemic (Figure 2).

Mismanagement of Interns and Junior Residents and a Demanding Competitive Work Environment

**Figure 3:**
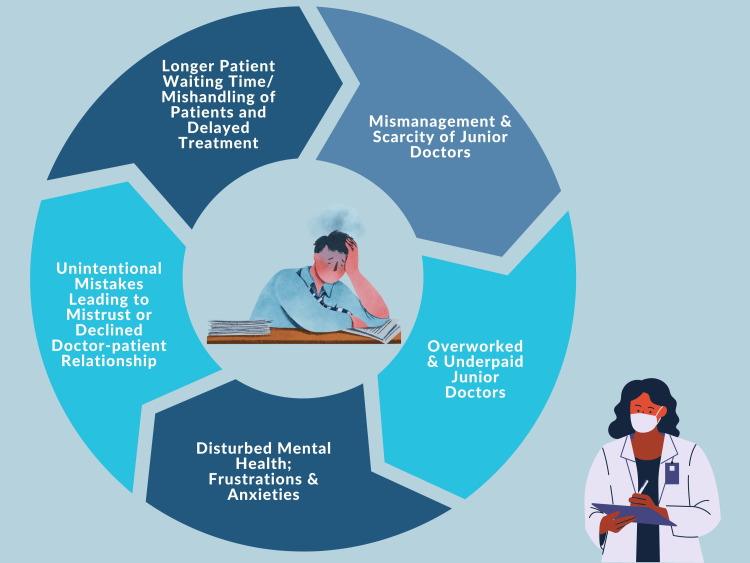
The vicious cycle of mismanagement of young doctors The illustration of this input has been created by the authors.

Interns spend their internships preparing for postgraduate (PG) entrance exams, which reduces their engagement with patients and practical knowledge as well. No national policy handles their stipend, job hours, or welfare. This mishandling results in a lack of work ethic, which is worsened by the pressure of PG entrance exams. Bullying, abuse, intimidation, slander, and gender-based humiliation by seniors make the workplace environment uncomfortable. With high patient turnover and limited resources, fresh graduates must manage patient care and exam preparation. This uneven work-life balance invariably affects the social lives of doctors. All these circumstances cause concern, forgetfulness, and lack of sleep or rest, which lead to therapeutic errors. It diminishes job satisfaction, self-confidence, and psychological anguish among professionals, which ultimately affects patient care [6,7]. Figure 3 describes these circumstances that create a vicious cycle leading to violence.

Overworked Healthcare Workers

Interns and residents who put in long hours are more exposed to violence than older doctors. Despite government instructions to work 48 hours per week with one day off and up to 12 hours per day, junior doctors are overworked [8]. Over the last two decades, surveys indicate that interns work more hours than residents, and surgical subspecialists tend to work the most, ranging from 35 to 120 hours per week on average [8]. A study done by Howard suggests that respondents believe that longer work hours negatively impact patient care and safety [9]. This causes long wait times and delays in treatment, which increase patient and attendee stress and worries, leading to violence [10]. Overburdening and following triage even in normal times induce patient mismanagement. A study conducted in a tertiary care hospital in New Delhi found that 73.5% of doctors mentioned long wait times as a cause of violence [11]. Multiple studies have discovered that violations of visiting hours and long wait times are the leading causes of violence toward healthcare professionals. Due to a lack of staff, relatives of patients helped the on-duty doctor during the second wave of the pandemic. Leaving patient attendants (and even patients) to undertake unpleasant chores accelerates viral infections and mortality into violence [6,12,13].

Underpaid Doctors

Many protests by doctors have called for higher residency and internship stipends [14]. Medical interns from government medical colleges (GMCs) across various Indian states are being paid a monthly stipend of rupees (INR) 11,000 (on average), but several private institutes across the nation are not following the same path. This low stipend impacts the quality of work management [15].

Inadequate Medical Infrastructure

India spends 1.3% of its gross domestic product (GDP) on health care, reflecting that it has never been a priority. Universal Health Coverage by National Health Policy suggests allocating 4% of GDP to health, while India plans to increase it to 2.5% by 2025 [16,17]. Currently, India ranks 145th out of 195 nations for healthcare access and quality. Overcrowding, long wait times, and substandard infrastructure result in multiple visits, delayed initial contact, poor emergency care, excessive referrals, and a hostile environment, which all contribute to violence. All these factors exposed the already crumbling healthcare system during the second wave of the pandemic [10,18-21].

Patient Out-of-Pocket Expenditure

In India, the government covers up to 33% of healthcare costs. Over 67% of healthcare costs are motivated by patients' mistaken belief that spending more money will save their lives, primarily in critically ill conditions [22]. Constantly low healthcare funds lead to a shortage of vital pharmaceuticals, causing physicians to ask patients to purchase medicines from other sources, putting patients' funds at risk. An estimated 60 million patients (about twice the population of Texas, USA) each year pay out of pocket for testing and medication, resulting in rising costs of therapy, vulnerability, unrealistic expectations, and financial stress [23]. These insecurities and stress among patient attendants result in greater vocal communication, abuse, and violence.

Lack of Medical Doctors

The Indian government estimates that there are around 10 lakh doctors in the country, both in the public and private sectors [24]. This number is not sufficient to meet the healthcare needs of India's population of 1.35 billion people. The shortage is particularly acute in rural areas, where access to healthcare is limited. The shortage of doctors in India is mainly due to the uneven distribution of healthcare resources, with a concentration of healthcare professionals in urban areas, leaving rural areas underserved. The lack of infrastructure, facilities, and training opportunities in rural areas exacerbates the issue. The shortage of doctors is putting a strain on the existing healthcare workforce, leading to stress and burnout among overworked doctors and nurses, which can cause a decline in the quality of care and a higher incidence of medical errors and adverse outcomes.

Poor Status or Lack of Standard Medical Education

Standardized medical examinations ensure that medical students acquire the knowledge and skills to become competent doctors. In India, the lack of standardization in testing across medical colleges poses serious implications for patient care [13]. The Medical Council of India (MCI) sets minimum standards for medical education, but there is significant variation in the quality of education across the country. The lack of standardization in testing leads to variability in the quality of doctors graduating from different colleges, which can result in inadequate patient care. Furthermore, it becomes difficult to evaluate the effectiveness of medical education programs without a common standard for comparison.

Private Health Care System

As healthcare in India becomes more commercialized, patients are increasingly being treated as consumers and hospitals as shops or establishments. Private hospitals are required to pay market prices and taxes, while government insurance policies often do not include private hospitals, which provide 80% of the country's healthcare services [25]. These factors force private hospitals to charge unreasonable prices and, in some cases, even raise facility treatment expenses. Additionally, the prevalence of fraud in the private sector further exacerbates the issue. Data from 2017 to 2018 indicates that only 37.2% of Indians had health insurance coverage, with 82% of urban residents lacking insurance altogether [26,27]. Furthermore, there is currently no community-wide health insurance program at government hospitals.

Lack of an Effective Grievance Redressal System in Government Hospitals

Government hospitals in India lack an effective grievance redressal system. Although a mechanism exists for relatives to express complaints, it often falls short of providing solutions. Doctors are left to handle the emotional responses of grieving relatives going through various stages of grief, including blame or condemnation towards doctors for a loved one's death [10]. Unpleasant medical events or the untimely death of a patient can also lead to mob mentality, with caregivers threatening doctors and damaging hospital property [6]. A robust grievance redressal system is essential to providing swift and effective solutions while ensuring the safety of doctors and hospital staff.

Underreporting of Cases: A Major Barrier

Workplace violence is a pervasive issue that affects employees across a wide range of industries and sectors. While many organizations have implemented strategies and programs to prevent WPV, underreporting incidents remains a major barrier to effectively addressing this issue [6,28]. In India, violence against doctors is a significant issue that is largely underreported. The fear of public, administrative, and law enforcement persecution is one of the reasons behind this trend. According to the study conducted by the Institute of Medicine and Law, only 52.1% of WPV incidents were reported to seniors and/or department heads, 32.8% to administrators, and 24.5% to the police. This suggests that a significant portion of WPV incidents go unreported, hindering the ability of healthcare organizations to address this issue. Moreover, the study found that only 9.9% of the study participants who reported an incident of violence were redressed adequately [4]. This highlights the need for healthcare organizations and other employers to take a more proactive approach to addressing WPV.

Doctor-Patient Mistrust: Causes and Consequences

Trust is the foundation of the doctor-patient relationship. However, in recent years, there has been a growing sense of mistrust between doctors and patients. This mistrust can have serious consequences for the quality of healthcare and patient outcomes.

Causes of mistrust: There are several factors that contribute to mistrust between doctors and patients. Patients now have access to vast amounts of health information, some of which may be inaccurate or misleading. This can lead to patients questioning their doctors' advice and recommendations and sometimes seeking alternative treatments that may not be effective or safe. Another factor is the increasing commercialization of healthcare. Patients may feel that their doctors are more interested in making money than providing quality care, particularly if they perceive that their doctor is recommending unnecessary treatments or procedures [29].

Consequences of mistrust: Mistrust between doctors and patients can have serious consequences. Patients who do not trust their doctors may be less likely to follow their recommendations, leading to poor health outcomes. They may also be more likely to seek out alternative treatments or delay seeking medical care, which can exacerbate their condition. Doctors, on the other hand, may become frustrated with patients who do not follow their advice or question their recommendations. This can make it more difficult for doctors to provide effective care and can lead to burnout and dissatisfaction with their profession.

The Psycho-Social Impact

Medical professionals are in distress. Most doctors describe insomnia, melancholy, worry, and an inability to visit patients without fear. An IMA study revealed that over 82.7% of Indian doctors feel stressed, with 46.3% citing fear of violence as the main cause. Around 62.8 percent of doctors are unable to treat their patients without any fear of being abused, and 24.2% fear being sued. When left to their own difficulties, doctors' morale decreases [1]. The detrimental impact of these episodes extends beyond doctors' physical and mental health to job performance, burnout, and the intention to leave, all of which can affect patient care quality. Uninformed civilians and overburdened doctors are forced to confront uncompromising authorities.

In extreme cases of WPV, healthcare workers strike to protest the authorities’ response. These massive strikes could result in lost work days, staffing shortages, and a load on the healthcare system [6,30]. Violence and stress often compound, establishing a vicious cycle [4,30]. Due to these circumstances, young doctors are moving abroad for better workplace security and better opportunities instead of staying in India, worsening the problem. It is estimated that anywhere from 20% to 50% of Indian healthcare workers intend on searching for employment overseas [31].

What can be done to address WPV against medical personnel

To reduce violence against medical professionals, better healthcare facilities, crisis management, employee training, and strict laws are needed. Recommendations include changing the curriculum, understanding violent patients, and educating patients and relatives. All stakeholders must work together to prevent this public health hazard.

What a Doctor Can Do

The doctor should know his/her limits and when to refer a patient to a more skilled colleague. They must be calm, and composed and know how to break bad news. Doctors should try to reduce patient wait times and inform patients well in advance in case of unavailability. Doctor-patient communication should be improved to bridge the gaps in information. The ailment, available treatment options, its side effects, alternatives to the indicated management plan, expected treatment duration, likely untreated outcome, overall prognosis, and financial consequences must be shared with patients and their families. Daily updates on the patient's status and video counseling with the family are important practices. Empathy and clear communication help reduce misunderstandings. Better and prompt communication with patients and their families can reduce inevitable violence and adverse events. In those potentially violent situations, doctors should watch for signs of hostility in a patient or their relative. One of the preventive strategies includes looking for STAMP, an acronym for the following red flags [32]: 1) Staring to intimidate, keeping continuous eye contact; 2) Tone and volume of voice, yelling, sarcastic and caustic replies; 3) Anxiety approaching vicious levels; 4) Mumbling suggests increasing frustration and anger; 5) Pacing around the room in agitation.

What a Hospital Can Do

The hospital's security needs efficient people and rapid communication with the police station. Developing a hospital security system equipped with alarm bells at critical areas to alert, closed-circuit cameras in public spaces, and practice exercises or mock drills to assess standard operating procedure (SOP) compliance are examples [10,16]. ​​​​​​ A live digital board showing the availability of ER and ICU beds should be put at the hospital's entrance to allow attendants to make a prior decision so they can transfer the patient to another hospital in time once primary care is delivered [13]. The hospital needs an effective complaint process for patients and professionals. Employees should be encouraged to call out any incident of any kind of abuse. Additionally, organizations should ensure that employees who report incidents are adequately supported and that effective measures are taken to address the root causes of WPV.

An accurate account of a violent incident can offer pertinent data for planning prevention and intervention reforms. The management should provide emotional assistance to counselors and social workers to reduce anxiety in a tense situation [10,16]. Improving patient satisfaction requires a transparent billing system and a grievance bureau. Hospitals and clinics should post patients' rights and duties along with the legal consequences of any abuse of healthcare workers. Posting signboards and banners informing patients and their attendants of the act and ordinances against violence, at some strategic locations would be a step in the right direction. Associations should coach doctors on etiquette, decorum, and ethics, along with anxiety alleviation techniques. Patients should be counseled on informed consent, emergency triage, and the difference between error and carelessness.

What the Government Can Do

**Figure 4:**
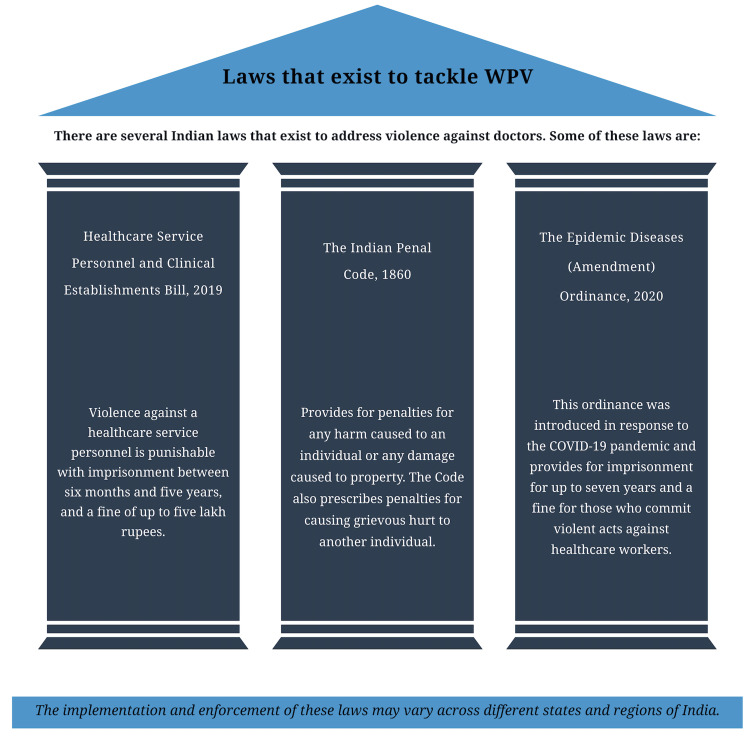
Existing laws in India that address violence against doctors This illustration of the data derived from an article written by Ram AB [33] has been created by the authors.

State governments should enforce strict rules on violence against doctors. Workshops, seminars, and other events should be conducted for doctors to familiarize them with the rules and laws they can rely on. Strengthening the legal framework and good national policies are needed to preserve doctors' rights. Violence against healthcare providers should be a punishable offense under the Indian Penal Code and Criminal Procedure Code [17]. Figure 4 describes the legal coverage doctors have against WPV at present in India.

The government must also improve hospital facilities and fill unfilled positions to address the personnel shortfall [16]. Skills enhancement for new recruits should be more focused. The COVID-19 pandemic has been a reality check for healthcare, and it revealed the dire need for an increase in the health budget, strong preventive medicine, and improved primary healthcare. By supplying medications, devices, and staff to primary and secondary care centers, many diseases can be treated at this level, allowing tertiary care doctors to focus on patients requiring specialized care. Mass education and greater access to primary healthcare are needed to avert violence against healthcare staff [7]. Medical schools can raise awareness of violence. Along with medical difficulties, they should educate students about better patient-doctor interactions, good communication, and empathy. Medical education should incorporate soft skills and communication qualities needed to sympathize with fearful patients [11,17,34-40].

Role of the Media

Media platforms must report responsibly, raise awareness, and facilitate constructive dialogue to prevent violence against doctors in India [13]. Both print and electronic media should report unbiasedly, without prejudice, unfairness, or fakery [7]. Social media, along with mainstream media, can encourage constructive dialogue between doctors, patients, and policymakers to address the root causes of violence. This can help put pressure on authorities to take timely action and form strict laws. Sensible reporting on healthcare can create a safer and more supportive environment for doctors to provide essential healthcare services.

What Society Can Do

Patients, their families, and society all share responsibility for preventing violence. Vandalism and violence in a hospital or clinic are crimes, and civilized society should have no space for any act of violence [41]. Social leaders must denounce violence against doctors and healthcare workers, and there should be awareness that medical costs can rise with technological improvements. Understanding the complexities of medical treatment, including diagnosis and uncertainties, is essential. Patients and their families should seek redress through the appropriate channels, such as senior doctors, grievance departments, or the legal system, instead of resorting to violence. They must be cognizant of the fact that the worldwide web can only provide information but not medical care.

## Conclusions

Violence against doctors jeopardizes patient care and safety. It requires radical changes in India's healthcare system. A combination of education, training, and policies can reduce the frequency and severity of incidents. Reforms from leaders with political will are required to stop this silent epidemic. It is crucial to recognize the vital role that doctors play and take concrete steps to protect them, ensuring quality healthcare for all. Healthcare organizations, law enforcement agencies, and policymakers must work together to create a safe and supportive environment for healthcare professionals. The increasing prevalence of such incidents highlights the urgent need for awareness, prevention, and response measures to address this multifaceted public health issue. This review lists some incidents of violence against healthcare professionals in India since 2021. While these are taken from various news media reports available online, they do not include every single incident that occurred. Adequate documentation of such incidents is the need of the hour to stop WPV against doctors and determine and address its root cause.

## Figures and Tables

**Figure 5:**
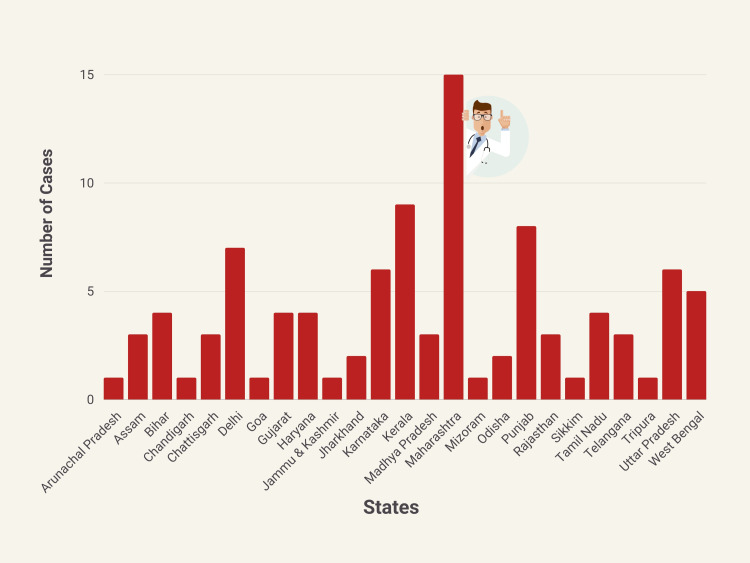
State-wise representation of WPV in India This bar chart created by the authors, represents the incidence of WPV against healthcare professionals (data collected from news articles) that took place from January 2021 to March 2023 across India. WPV: Workplace violence

## Appendices

This supplementary appendix includes Tables 1-3 featuring the date, place, state, and a brief summary of WPV against healthcare professionals reported (scroll down for links on reportage) in India between 2021 and 2023. Table 4 lists the rate of incidence of WPV against medical professionals in different Indian states while Figure 5 is a graphical representation of it. 

**Table 1:** Timeline and details of WPV against medical professionals in India that took place in 2021 WPV: Workplace violence, IMA: Indian Medical Association

WPV against healthcare professionals in 2021				
S.no	Date	Place	State	Incident
1	February 12	Private Clinic, Harni, Vadodara	Gujarat	A urologist was physically assaulted by a relative/friend of a patient who was operated on by him four months ago
2	February 17	Pokhran Government Hospital, Jaisalmer	Rajasthan	A government doctor suffered grave injuries and a fracture in his hand after the family of a patient assaulted him when he allegedly denied complying with their demand of going to the hospital to examine the patient for a second time
3	February 23	Dalton Ganj (Medini Nagar)	Jharkhand	On-duty resident doctor was assaulted by the kin of a dead minor; his colleagues demand action
4	February 23	Bhim Rao Ambedkar Hospital, Raipur	Chhattisgarh	A doctor was thrashed and assaulted after a verbal spat by a jail guard who was irked over the delay in the MRI scanning of an ill prisoner
5	March 13	Hindu Rao Hospital, New Delhi	Delhi	After the death of a rickshaw puller during treatment the family and some of his kin vandalized the hospital as well as attacked the doctors in the emergency department
6	March 13	Patna	Bihar	Inspector assaulted a doctor alleging improper plaster on son's leg
7	March 14	Aster CMI Hospital, Hebbal, Bengaluru	Karnataka	The relatives of a three-year-old patient allegedly slapped a pediatrician in the pediatric intensive care unit, threatened him, and even left without signing the discharge documents and paying the bill partly
8	March 15	Bandra, Mumbai	Maharashtra	A senior on-duty doctor was manhandled after an altercation broke out with the patient for suggesting he undergo a COVID-19 test as he had a high temperature and had recently traveled from another state
9	March 15	Amritsar Civil Hospital, Amritsar	Punjab	A medical officer sustained a serious bullet injury after he got caught in the middle of open firing between two groups
10	April 5	Pune	Maharashtra	An elderly MBBS doctor was abused, beaten black and blue by society worker
11	April 8	Amritsar	Punjab	Doctor shot and robbed of his car at gunpoint
12	April 11	Chahut village, Dhudhan Sadhan block Patiala	Punjab	A medical officer was beaten up during the cremation of a COVID-19 victim by villagers for following COVID protocols. Timely intervention by a few villagers helped the doctor to flee to safety
13	April 11	Bhopal	Madhya Pradesh	A woman doctor and nurse were attacked and held hostage at a COVID patient's house
14	April 17	Hisar	Haryana	An on-duty doctor was abused by the kin of an elderly patient who passed away due to COVID-19
15	April 27	Apollo Hospital, Sarita Vihar, New Delhi	Delhi	The family of a woman, who allegedly died due to COVID-19, attacked the staff members, including nurses and doctors, vandalizing the hospital properties for not allotting a bed to the patient on time
16	May 3	Itanagar	Arunachal Pradesh	Mob lynching of two doctors; medical fraternity up in arms
17	May 6	Dadri Community Health Centre (CHC), Noida	Uttar Pradesh	An on-duty doctor serving at the emergency ward was locked up inside an ambulance by the angry relatives of a patient who died allegedly without being attended by the doctors
18	May 13	Belagavi, Karnataka	Karnataka	Mob attacks doctors, nurse for treating COVID patients
19	May 14	District Hospital in Mavelikara in Alappuzha, Thiruvananthapuram, Kerala.	Kerala	A COVID-19 patient was brought dead to the hospital. The patient’s son allegedly slapped a surgeon working in the night shift, alleging that his mother died because of the latter’s negligence.
20	May 23	VIMS Hospital, Ballari	Karnataka	A lady duty doctor was allegedly attacked by a relative of a Covid-19 patient.
21	May 25	Matiyari Trisection, Chinhat, Lucknow	Uttar Pradesh	A doctor and owner of a hospital was shot barely 300 meters away from his house by some miscreants who came in an SUV
22	May 27	Virinchi Hospitals, Hyderabad	Telangana	Doctor assaulted, hospital ransacked by a mob of 16 people
23	May 27	Greater Noida	Uttar Pradesh	Miscreants opened fire and threatened a doctor for being denied entry into the clinic without masks
24	May 31	Palghar district, Thane	Maharashtra	A team of doctors and healthcare workers, visiting to spread awareness about COVID-19 and its vaccination were brutally assaulted and manhandled by the villagers
25	May 31	Tarikere town, Chikkamagaluru	Karnataka	A 50-year-old pediatric doctor was severely injured in an attack by four people, including a minor who was a patient's relative
26	June 01	Udali Covid Care Centre, Hojai, Guwahati	Assam	A young doctor and other medical staff were thrashed by a mob of 40 people following the death of an aged COVID-positive (brought in with a low SpO2 and urinary retention) patient.
27	June 14	Fortis Hospital, Bengaluru	Karnataka	A nurse and a senior doctor were brutally assaulted by the relatives of a COVID-19 patient who passed away after being on a ventilator for 35 days
28	June 25	Sion Hospital, Mumbai	Maharashtra	An on-duty resident doctor was assaulted by the relatives of a patient
29	June 28	Gujarat Medical Education and Research Society Hospital, Sola, Ahmedabad	Gujarat	A resident doctor was slapped and assaulted by two men
30	July 1, on the eve of Doctor's Day	Gautam Buddh Nagar	Uttar Pradesh	An angry mob brutally assaulted two junior resident doctors (from AIIMS, Delhi) with iron rods causing several head injuries, alleging that doctors are infecting the locality with Coronavirus
31	July 11	Sub District Hospital (SDH) Tangmarg, Baramulla	Jammu & Kashmir	Two doctors and two staff members on COVID duty were physically assaulted by a group of tourists who were allegedly demanding a negative COVID test report, which is required to visit some tourist destinations.
32	July 20	Balipatna Community Health Centre (CHC), Bhubaneswar	Odisha	Doctor and nurse were assaulted at CHC by a patient's kin who were angry over the delay in administering an injection
33	July 21	Raichur Institute of Medical Sciences (RIMS)	Karnataka	Attack on two on-duty intern doctors by the relatives of a deceased patient
34	July 23	Block Primary Health Centre (BPHC), Murshidabad	West Bengal	On duty doctor, two nurses were assaulted by patient's relatives
35	July 26	Government Chest Hospital, Hyderabad	Telangana	A junior doctor was assaulted by a COVID-19 patient's attendants
36	August 14	Gokulam Medical College, Attinga, Thiruvananthapuram	Kerala	A female doctor was attacked with slippers by the patient and attendant
37	August 30	PGIMS Rohtak	Haryana	A doctor was abused and assaulted by a female attendant of the patient
38	September 15	Gurugram	Haryana	A hospital owner was assaulted by three men
39	September 27	Patna	Bihar	A private doctor was allegedly assaulted, and his clinic was vandalized by the patient’s kin over signing a consent form; IMA demands action
40	November 9	Nightingale Hospital, Silchar	Assam	Doctor physically assaulted; hospital ransacked after a pregnant woman's death
41	November 26	Shahid Nirmal Mahto Memorial Medical College and Hospital Dhanbad	Bihar	Female junior doctor was assaulted by a mob of attendants
42	December 14	STNM Hospital, Gangtok	Sikkim	Cardiologist was attacked with a knife in Sikkim and succumbed to injuries
43	December 24	Patna Medical College and Hospital	Bihar	Duty doctors were beaten by attendants of a newborn for lack of facilities
44	December 26	Dombivli, Thane	Maharashtra	Doctor assaulted by kin of a nine-month-old baby that died in a hospital

**Table 2:** Timeline and details of WPV against medical professionals in India that took place in 2022 WPV: Workplace violence, PPH: Postpartum hemorrhage, DOA: Dead on arrival

WPV against healthcare professionals in 2022				
S.no	Date	Place	State	Incident
1	January 5	Private multi-specialty hospital, Madurai	Tamil Nadu	Two relatives of a patient in the emergency room first verbally abused medical staff including doctors and nurses, and prevented them from giving any treatment. Then physically assaulted and threatened the doctor.
2	January 23	Private clinic, Bhayandar (west), Thane	Maharashtra	A man posing as a patient brutally attacked a female doctor inside her clinic
3	January 27	Mankapur, Nagpur	Maharashtra	A mob of 30 people assaulted a senior physician and vandalized a private hospital
4	February 7	RTR Memorial Hospital, Dwarka, Delhi	Delhi	Resident doctor was shot by unknown assailants outside the hospital
5	February 16	Private Clinic, Adarsh Nagar, New Delhi	Delhi	A 50-year-old lady doctor was robbed at gunpoint
6.	February 21	Postgraduate Institute of Medical Education and Research, Chandigarh	Chandigarh	Resident doctor was physically assaulted by a grieving attendant
7	March 23	Madhuram Hospital, Rajkot	Gujarat	On-duty doctor was assaulted by patient kin
8	March 29	Lalsot, Dausa	Rajasthan	Booked for murder and medical negligence after the death of a patient during childbirth, a distressed gynecologist committed suicide at the hospital
9	April 01	Chhattisgarh Institute of Medical Sciences (CIMS), Raipur	Chhattisgarh	Patient brutally assaulted a medical officer due to a delay in the treatment
10	April 08	Tisgaon, Ahmednagar	Maharashtra	Gynecologist was assaulted over PPH death
11	April 09	Howrah District Hospital, Kolkata	West Bengal	Doctors, nurses, and hospital staff were brutally assaulted by a patient's family; hospital vandalized; a doctor suffered grievous bodily harm (dislocation of right shoulder)
12	April 15	Central Hospital, Tilak Nagar, Delhi West	Delhi	A doctor in the ICU was attacked by a violent patient with a pair of scissors
13	April 18	State General Hospital, Birpara, Alipurduar	West Bengal	On-duty pediatrician was assaulted by the patient's father for delay in examining the latter's child who was suffering from gastroenteritis
14	April 21	Colvale Jail, Panaji	Goa	Medical officer was assaulted by an inmate while performing his OPD dispensary duties at Colvale jail
15	May 04	Manikpur Primary Health Center, Dhalai	Tripura	Following the death of two minor patients, a mob of villagers vandalized the hospital and allegedly thrashed an on-duty doctor
16	May 18	Kalawati Saran Children's Hospital, New Delhi	Delhi	Doctor was assaulted for refusing to admit DOA patient
17	May 30	Duldula Community Health Center, Jashpur, Raipur	Chhattisgarh	Medical officer was manhandled and abused during an inspection by an MLA
18	June 02	Neendakara Taluk Hospital, Kollam	Kerala	Medical staff were attacked
19	June 24	Puducherry	Tamil Nadu	Medical intern was assaulted
20	July 18	Civil Hospital, Ferozepur	Punjab	Two on-duty pediatricians were attacked and harassed
21	August 06	GMCH, Aurangabad	Maharashtra	Resident doctor was assaulted by relatives of a patient
22	August 17	Howrah, Kolkata	West Bengal	A doctor was brutally attacked by a mob of drunk goons at his residence after he protested against drinking alcohol
23	August 21	Aizawl	Mizoram	Dermatologist attacked by Mizoram CM’s daughter indicating errors of VIP culture
24	August 28	Rourkela Government Hospital (RGH)	Odisha	Staff nurse were assaulted by relatives alleging medical negligence
25	August 28	Nashik Hospital, Nashik	Maharashtra	Doctor was attacked by relatives over the issue of providing reports
26	October 29	GMCH, Shahjahanpur	Uttar Pradesh	A politician pointed a gun and threatened an on-duty doctor in an emergency
27	September 03	District hospital, Panna	Madhya Pradesh	A civil surgeon was attacked by an accident victim's family
28	September 14	GRMC, Gajra Raja Medical College, Gwalior	Madhya Pradesh	Policemen beat up a medical student
29	September 26	DY Patil Hospital, Pimpri, Pune	Maharashtra	An assistant medical officer was assaulted over a dispute regarding surgery
30	September 30	Private hospital, Kozhikode	Kerala	Three students manhandled a doctor during a medical checkup
31	October 10	Osmania General Hospital, Hyderabad	Telangana	Junior doctors were attacked
32	October 12	Bai Rukminibai Hospital, Thane	Maharashtra	Doctor was attacked by a drunk man
33	October 28	General Hospital	Kerala	Doctor was assaulted for forcefully suggesting a patient be admitted
34	October 28	BRD Medical College and Hospital, Gorakhpur Medical College	Uttar Pradesh	Six healthcare staff including doctors were grievously injured
35	November 10	Private Clinic Ranjit Nagar, Ludhiana	Punjab	Doctor was attacked at a clinic by four men; cash and mobile phone stolen
36	November 13	Phagwara Civil Hospital, Phagwara	Punjab	Emergency doctor was attacked after the death of a teenager in a train accident
37	November 23	MBS Hospital, Kota	Rajasthan	Doctor was slapped and attacked with a chair by relatives of a dead patient
38	November 30	MCH, Thiruvananthapuram	Kerala	Female neurosurgeon was attacked over the death of a patient
39	December 05	SSKM Medical College and Hospital, Kolkata	West Bengal	Trauma care doctors were assaulted, and the hospital vandalized after a patient’s death
40	December 16	Lokmanya Tilak Municipal General Hospital, Sion, Mumbai	Maharashtra	Resident doctor was slapped by a constable over the discharge of a patient

**Table 3:** Timeline and details of WPV against medical professionals in India that took place in 2023 WPV: Workplace violence

WPV against healthcare professionals in 2023				
S.no.	Date	Place	State	Incident
1	January 5	Shri Vasantrao Naik Government Medical College (GMC) Yavatmal Hospital, Yavatmal	Maharashtra	Two resident doctors were attacked by a patient with a knife
02	January 8	Tirunelveli Medical College and Hospital (TVMCH), Tirunelveli	Tamil Nadu	A house surgeon was attacked after the death of a patient
03	January 8	Private Hospital, Kochi	Kerala	A man slapped a doctor for merely touching a patient in the casualty department
04	January 9	MCH, Thiruvananthapuram	Kerala	Female nurse was abused and manhandled over delay in administering drip
05	January 14	Private Nursing Home, Bathinda	Punjab	Doctor was shot by two masked men inside a private nursing home
06	January 21	District Hospital, Palghar	Maharashtra	Doctor was assaulted, hospital vandalized over a billing dispute
07	January 31	Government Rajindra Hospital, Patiala	Punjab	Third-year ENT resident suffered nose fracture after brutal attack
08	February 11	Rajiv Gandhi Hospital, Chennai	Tamil Nadu	Postgraduate doctor was assaulted by relatives of a deceased patient
09	February 22	District Hospital, Dibrugarh	Assam	Medical intern was brutally attacked and grievously injured by two men
10	February 28	Gulmohar Hospital, Ranchi	Jharkhand	Mob entered an orthopedician’s home and attacked with a rod
11	March 2	Civil Hospital, Nashik	Maharashtra	Nursing staff (including a class 4 employee) were assaulted by a family when the patient's husband was asked to leave the ward during cleaning
12	March 4	Fathima Hospital, Kozhikode	Kerala	Senior cardiologist was assaulted over a delay in treatment
13	March 5	Babu Jagjivan Ram Govt. Hospital, New Delhi	Delhi	Two night-duty doctors and staff were attacked by the kin over a delay in attending to the patient
14	March 12	Badshah Khan Civil Hospital, Faridabad	Haryana	40-year-old doctor in emergency room was assaulted over delay in treatment
15	March 15	GK General Hospital, Bhuj, Rajkot	Gujarat	Two men attacked a doctor in the emergency department over a dispute over treatment

**Table 4:** State-wise distribution of WPV incidence in India Listed are all the reported incidents (compiled from news articles) of WPV in different states of India from 1st January 2021 to 31st March 2023. WPV: Workplace violence

S.no	States	Number of cases
1	Arunachal Pradesh	1
2	Assam	3
3	Bihar	4
4	Chandigarh	1
5	Chattisgarh	3
6	Delhi	7
7	Goa	1
8	Gujarat	4
9	Haryana	4
10	Jammu & Kashmir	1
11	Jharkhand	2
12	Karnataka	6
13	Kerala	9
14	Madhya Pradesh	3
15	Maharashtra	15
16	Mizoram	1
17	Odisha	2
18	Punjab	8
19	Rajasthan	3
20	Sikkim	1
21	Tamil Nadu	4
22	Telangana	3
23	Tripura	1
23	Uttar Pradesh	6
24	West Bengal	5
Total no. of incidents		99

Reports of WPV against medical personnel in India from 2021 to 2023

2021

1. https://medicaldialogues.in/news/health/doctors/gujarat-doctors-suspend-opd-services-after-patients-kin-assaults-urologist-74485

2. https://medicaldialogues.in/news/health/doctors/rajasthan-govt-doctor-suffers-fracture-in-attack-by-patients-kin-2-arrested-74709

3. https://medicaldialogues.in/news/health/doctors/on-duty-resident-doctor-assaulted-by-kin-of-dead-minor-colleagues-demand-action-74894

4. https://www.hindustantimes.com/india-news/video-of-jail-guard-assaulting-chhattisgarh-doctor-goes-viral-doctors-on-strike-101614060118875.html

5. https://www.indiatoday.in/cities/delhi/story/delhi-hindu-rao-hospital-doctors-demand-fir-against-patient-s-kin-who-attacked-them-1778872-2021-03-13

6. https://medicaldialogues.in/news/health/doctors/patna-inspector-assaults-doctor-alleging-improper-plaster-on-sons-leg-arrested-75488

7. https://medicaldialogues.in/news/health/doctors/pediatrician-slapped-threatened-by-kin-after-death-of-3-year-old-patient-in-aster-cmi-hospital-75508

8. https://medicaldialogues.in/news/health/doctors/man-arrested-for-manhandling-on-duty-bmc-doctor-after-altercation-over-covid-19-testing-75546

9. https://www.hindustantimes.com/cities/others/doctor-injured-in-firing-during-clash-at-amritsar-civil-hospital-101615749889392.html

10. https://medicaldialogues.in/news/health/doctors/elderly-mbbs-doctor-abused-beaten-blue-and-black-by-society-worker-76447

11. https://medicaldialogues.in/news/health/doctors/traumatised-after-being-assaulted-by-villagers-doctor-decides-to-quit-profession-76457

12. https://medicaldialogues.in/news/health/doctors/traumatised-after-being-assaulted-by-villagers-doctor-decides-to-quit-profession-76457

13. https://medicaldialogues.in/state-news/madhya-pradesh/mp-woman-doctor-nurse-attacked-held-hostage-at-covid-patients-house-family-booked-76599 

14. https://medicaldialogues.in/news/health/doctors/haryana-duty-doctor-abused-after-death-of-covid-patient-2-booked-76973

15. https://www.tribuneindia.com/news/delhi/delhi-covid-19-patient-fails-to-get-bed-dies-family-attacks-hospital-staff-244988

16. https://medicaldialogues.in/news/health/doctors/stir-over-attempted-mob-lynching-of-2-arunachal-doctors-state-ima-colleagues-go-on-strike-77438

17. https://medicaldialogues.in/news/health/doctors/up-angry-kin-lock-up-on-duty-doctor-in-ambulance-after-patient-death-at-noida-hospital-77315

18. https://medicaldialogues.in/news/health/medical-organization/karnataka-mob-attacks-doctors-nurse-for-treating-covid-patients-state-ima-threatens-stir-77567

19. https://medicaldialogues.in/state-news/kerala/on-duty-surgeon-assaulted-by-kerala-police-personnel-after-covid-patient-death-78603

20. https://www.newindianexpress.com/states/karnataka/2021/may/23/karnataka-covid-patients-kin-assault-lady-doctor-in-ballaris-vims-hospital-arrested-2306589.html

21. https://timesofindia.indiatimes.com/city/lucknow/lucknow-doctor-shot-at-in-chinhat-critical/articleshow/82963217.cms

22. https://timesofindia.indiatimes.com/city/hyderabad/16-held-for-attacking-doc-ransacking-hosp/articleshow/83019010.cms

23. https://medicaldialogues.in/news/health/doctors/not-allowed-to-enter-clinic-without-mask-up-men-open-fire-threaten-doctor-78054

24. https://medicaldialogues.in/state-news/maharashtra/maha-covid-duty-doctors-healthcare-workers-attacked-by-villagers-in-palghar-2-held-78293

25. https://www.indiatoday.in/india/story/karnataka-doctor-cycling-home-for-lunch-brutally-attacked-over-6-year-old-dengue-patient-s-death-1810 106-2021-06-02

26. https://www.tribuneindia.com/news/nation/video-of-assam-doctor-being-brutally-thrashed-by-covid-victims-family-surfaces-online-262264

27. https://www.thenewsminute.com/article/staff-bengalurus-fortis-hospital-protest-after-assault-doctor-and-nurse-150654

28. https://medicaldialogues.in/state-news/maharashtra/violence-against-doctors-sion-hospital-residents-resume-duties-after-safety-assurance-dean-releases-provision-for-institutional-fir-79197

29. https://indianexpress.com/article/cities/ahmedabad/gujarat-resident-doctor-of-sola-hospital-assaulted-7380176/

30. https://timesofindia.indiatimes.com/city/delhi/two-doctors-attacked-in-delhis-gautam-nagar-over-covid-19-spread/articleshow/84012751.cms

31. https://timesofindia.indiatimes.com/city/srinagar/tourists-attacked-doctors-wanted-negative-covid-19-test-report-to-visit-gulmarg/articleshow/84216392.cms

32. https://www.orissapost.com/doctor-nurse-assaulted-at-balipatna-chc-in-khurda-district/

33. https://medicaldialogues.in/state-news/karnataka/rims-medicos-protest-against-alleged-assault-on-on-duty-intern-doctors-by-deceased-patients-kin-79990

34. https://medicaldialogues.in/state-news/west-bengal/on-duty-doctor-2-nurses-assaulted-by-patients-relatives-5-arrested-80092

35. https://medicaldialogues.in/news/health/doctors/telangana-covid-patients-relatives-assault-doctor-at-chest-hospital-colleagues-boycott-duties-80169

36. https://medicaldialogues.in/state-news/kerala/kerala-female-doctor-attacked-with-slippers-by-patient-attendant-80883

37. https://medicaldialogues.in/state-news/haryana/pgims-rohtak-doctor-abused-assaulted-by-female-attendant-of-patient-81439

38. https://medicaldialogues.in/news/health/doctors/gurugram-hospital-owner-assaulted-by-3-men-trio-booked-82045

39. https://medicaldialogues.in/news/health/doctors/doctor-beaten-up-by-patient-kin-over-signing-consent-form-ima-demands-action-82569

40. https://medicaldialogues.in/state-news/assam/doctor-beaten-up-hospital-ransacked-after-pregnant-woman-death-ima-demands-action-84265

41. https://medicaldialogues.in/news/health/doctors/over-135-doctors-boycott-duty-at-dhanbad-hospital-after-mob-attacks-female-medico-85015

42. https://medicaldialogues.in/news/health/doctors/cardiologist-injured-in-brutal-knife-attack-dies-86361

43. https://medicaldialogues.in/news/health/doctors/attendants-of-newborn-beat-duty-doctors-in-protest-against-lack-of-facilities-at-pmch-86363

44. https://medicaldialogues.in/news/health/doctors/kin-assault-doctor-after-9-month-old-baby-dies-in-hospital-4-booked-86393

2022

1. https://medicaldialogues.in/news/health/doctors/2-held-for-assaulting-doctor-medical-staff-in-madurai-hospital-86887

2. https://medicaldialogues.in/news/health/doctors/man-posing-as-patient-brutally-attacks-woman-doctor-inside-her-clinic-probe-launched-87618

3. https://medicaldialogues.in/news/health/doctors/mob-brutally-beats-up-doctor-vandalizes-nagpur-hospital-7-booked-87799

4. https://medicaldialogues.in/news/health/doctors/resident-doctor-shot-by-unknown-assailants-outside-rtr-memorial-hospital-88257

5. https://medicaldialogues.in/news/health/doctors/four-arrested-for-robbing-delhi-doctor-at-gunpoint-88818

6. https://medicaldialogues.in/state-news/chandigarh/pgi-chandigarh-doctors-see-red-after-colleague-slapped-on-patient-demise-89083

7. https://medicaldialogues.in/news/health/doctors/madhuram-hospital-on-duty-doctor-assaulted-by-patient-kin-3-arrested-90438

8. https://medicaldialogues.in/news/health/doctors/booked-for-murder-after-patient-death-distressed-gynecologist-commits-suicide-in-rajasthan-90654

9. https://medicaldialogues.in/news/health/doctors/chhattisgarh-institute-of-medical-sciences-patient-brutally-assaults-medical-officer-accused-under-arrest-90996

10. https://medicaldialogues.in/news/health/doctors/maharashtra-gynaecologist-becomes-yet-another-pph-victim-severely-beaten-up-after-patient-death-91235 

11. https://medicaldialogues.in/news/health/doctors/violence-continues-howrah-district-hospital-doctors-brutally-assaulted-by-patient-family-hospital-vandalised-91307

12. https://medicaldialogues.in/news/health/doctors/delhi-govt-doctor-allegedly-attacked-with-scissors-91507

13. https://medicaldialogues.in/news/health/doctors/on-duty-paediatrician-assaulted-by-patients-father-demanding-immediate-attention-91721

14. https://medicaldialogues.in/news/health/doctors/goa-shocker-doctor-assaulted-in-jail-91809

15. https://medicaldialogues.in/news/health/doctors/tripura-two-minor-siblings-dies-on-duty-doctor-allegedly-thrashed-by-mob-92473

16. https://medicaldialogues.in/news/health/hospital-diagnostics/lhmc-doctor-assaulted-for-refusing-to-admit-brought-dead-patient-93169

17. https://medicaldialogues.in/state-news/chattisgarh/chattisgarh-doctors-bodies-condemn-mo-assault-during-mla-inspection-93870

18. https://medicaldialogues.in/news/health/doctors/kerala-three-held-for-attacking-doctor-medical-staff-in-neendakara-hospital-94967

19. https://medicaldialogues.in/news/health/doctors/puducherry-200-intern-doctors-boycott-duty-to-condemn-assault-on-colleague-95244

20. https://medicaldialogues.in/news/health/doctors/punjab-two-on-duty-paediatricians-attacked-in-ferozepur-civil-hospital-opd-services-hit-96169

21. https://medicaldialogues.in/news/health/doctors/350-gmch-aurangabad-resident-doctors-stage-protest-to-condemn-assault-on-colleague-97163

22. https://medicaldialogues.in/news/health/doctors/west-bengal-doctor-brutally-thrashed-by-drunk-goons-probe-on-97696

23. https://medicaldialogues.in/news/health/doctors/vip-culture-mizoram-cms-daughter-hits-dermatologist-father-tenders-apology-after-severe-backlash-from-doctors-97873

24. https://medicaldialogues.in/news/health/hospital-diagnostics/odisha-rourkela-hospital-staff-nurse-assaulted-by-kin-after-patients-death-colleagues-condemn-attack-98360

25. https://medicaldialogues.in/news/health/doctors/brutal-assault-patients-relatives-thrash-nashik-doctor-vandalize-hospital-98357

26. https://medicaldialogues.in/news/health/doctors/up-bjp-leader-booked-for-threatening-govt-doctor-101547

27. https://medicaldialogues.in/news/health/doctors/mp-civil-surgeon-attacked-by-patients-kin-doctors-condemn-assault-98936

28. https://medicaldialogues.in/state-news/madhya-pradesh/policemen-allegedly-beat-up-mbbs-students-after-altercation-mp-juda-demands-action-threatens-strike-99190

29. https://medicaldialogues.in/news/health/doctors/maha-dy-patil-hospital-doctor-assaulted-by-96-year-old-patients-kin-99702

30. https://medicaldialogues.in/news/health/doctors/kerala-three-students-arrested-for-assaulting-doctor-100054

31. https://medicaldialogues.in/state-news/telangana/telangana-osmania-general-hospital-junior-doctors-stage-protest-to-condemn-assault-seek-security-100541

32. https://medicaldialogues.in/news/health/doctors/man-arrested-for-assaulting-thane-doctor-creating-ruckus-in-hospital-100648

33. https://medicaldialogues.in/news/health/doctors/attack-on-doctor-kerala-minister-says-attacks-against-health-workers-cannot-be-accepted-101478

34. https://tinyurl.com/bdbuwyk

35. https://medicaldialogues.in/news/health/doctors/ludhiana-doctor-assaulted-robbed-of-cash-4-booked-102131

36. https://medicaldialogues.in/news/health/doctors/punjab-phagwara-civil-hospital-doctors-stage-protest-to-condemn-assault-on-colleague-by-kin-of-deceased-patient-102266

37. https://medicaldialogues.in/news/health/doctors/resident-doctors-go-on-strike-after-colleague-slapped-attacked-with-chair-by-kin-of-deceased-patient-102923

38. https://medicaldialogues.in/news/health/doctors/assault-on-mch-female-neurosurgeon-only-one-bystander-for-inpatients-to-be-allowed-in-medical-colleges-103252

39. https://medicaldialogues.in/news/health/doctors/kolkata-sskm-junior-doctors-attacked-hospital-vandalised-after-patients-death-103594 

40. https://tinyurl.com/3tdb8ke4

2023

1. https://tinyurl.com/3jzhdnh6

2. https://medicaldialogues.in/news/health/doctors/tn-kin-assaults-house-surgeon-of-tirunelveli-medical-college-hospital-after-patients-death-105374 

3. https://medicaldialogues.in/news/health/medico-legal/man-slaps-doctor-for-merely-touching-wife-for-the-purpose-of-medical-examination-   denied-bail-107848 

4. https://medicaldialogues.in/news/health/hospital-diagnostics/kerala-mch-nurse-manhandled-by-patient-bystander-over-delay-in-administering-drip-bystander-arrested-105407

5. https://tinyurl.com/3wfd3jaz

6. https://medicaldialogues.in/news/health/doctors/palghar-doctor-attacked-hospital-vandalised-over-not-discounting-patient-bill-9-arrested-106032 

7. https://medicaldialogues.in/news/health/doctors/3rd-year-ms-ent-brutally-attacked-by-patient-attendants-at-gmc-patiala-suffers-nose-fracture-106429

8. https://tinyurl.com/3jtraz4b

9. https://tinyurl.com/ywnf7v44

10. https://medicaldialogues.in/news/health/doctors/are-doctors-not-even-safe-in-their-own-homes-miscreant-enters-orthopedicians-house-attacks-on-head-with-rod-107822 

11. https://tinyurl.com/42wvmx6u

12. https://medicaldialogues.in/news/health/attack-on-senior-cardiologist-court-grants-bail-to-five-accused-in-kozhikode-108945

13. https://medicaldialogues.in/news/health/doctors/violence-yet-again-two-on-duty-doctors-staff-attacked-by-patients-kin-for-delay-in-attending-to-patient-108144  

 14. https://medicaldialogues.in/news/health/doctors/faridabad-viral-video-40-year-old-doctor-on-emergency-duty-attacked-for-alleged-delay-in-treatment-3-arrested-108419

15. https://medicaldialogues.in/news/health/doctors/doctor-thrashed-by-patients-family-over-treatment-in-emergency-ward-of-bhuj-hospital-2-booked-108701
